# Many-Scale Investigations of Deformation Behavior of Polycrystalline Composites: II—Micro-Macro Simultaneous FE and Discrete Dislocation Dynamics Simulation

**DOI:** 10.3390/ma15082852

**Published:** 2022-04-13

**Authors:** Yanling Schneider, Dennis-Michael Rapp, Yifang Yang, Werner Wasserbäch, Siegfried Schmauder

**Affiliations:** 1 Institute for Materials Testing, Materials Science and Strength of Materials (IMWF), University of Stuttgart, Pfaffenwaldring 32, D-70569 Stuttgart, Germany; dennis-micheal.rapp@imwf.uni-stuttgart.de (D.-M.R.); yang-1217@outlook.com (Y.Y.); siegfried.schmauder@imwf.uni-stuttgart.de (S.S.); 2 Institute of Materials Science, University of Stuttgart, Heisenbergstraße 3, D-70569 Stuttgart, Germany; werner.wasserbaech@imw.uni-stuttgart.de

**Keywords:** many-scale simulation, crystal plasticity, local yield stress, texture, Σ3-twins effect, dislocation mechanisms, discrete dislocation dynamics

## Abstract

The current work numerically investigates commercial polycrystalline Ag/17vol.%SnO${}_{2}$ composite tensile deformation behavior with available experimental data. Such composites are useful for electric contacts and have a highly textured initial material status after hot extrusion. Experimentally, the initial sharp fiber texture and the number of Σ3-twins were reduced due to tensile loading. The local inhomogeneous distribution of hardness and Young’s modulus gradually decreased from nanoindentation tests, approaching global homogeneity. Many-scale simulations, including micro-macro simultaneous finite element (FE) and discrete dislocation dynamics (DDD) simulations, were performed. Deformation mechanisms on the microscale are fundamental since they link those on the macro- and nanoscale. This work emphasizes micromechanical deformation behavior. Such FE calculations applied with crystal plasticity can predict local feature evolutions in detail, such as texture, morphology, and stress flow in individual grains. To avoid the negative influence of boundary conditions (BCs) on the result accuracy, BCs are given on the macrostructure, i.e., the microstructure is free of BCs. The particular type of 3D simulation, axisymmetry, is preferred, in which a 2D real microstructural cutout with 513 Ag grains is applied. From FE results, Σ3-twins strongly rotated to the loading direction (twins disappear), which, possibly, caused other grains to rotate away from the loading direction. The DDD simulation treats the dislocations as discrete lines and can predict the resolved shear stress (RSS) inside one grain with dependence on various features as dislocation density and lattice orientation. The RSS can act as the link between the FE and DDD predictions.

## 1. Introduction

To improve metallic material performance and develop new ones, it is meaningful to investigate their physical and mechanical deformation behaviors. Any specific material behavior is a result of coupled mechanisms on different scales. To a great extent, the macroscopic material behavior depends on local features, such as local anisotropy, inhomogeneity, and micromorphology. The microscale acts as a bridge, linking the material deformation mechanisms and properties on the nano- and macroscale. Finite element (FE) simulation is the most important tool for investigating continuum-scale material deformation behaviors numerically. Constitutive modeling used in FE simulation is powerful to set up the relationships between the global material properties and the underlying local deformation mechanisms [1].

Many metallic alloys and composites have polycrystalline microstructures. Besides other local features, the crystal orientation evolution, grain interaction, and microstructural morphology influence the overall material properties. Crystal plasticity (CP) theories are preferred to better describe such micromechanisms. CP modeling considers grain orientation, individual slip system, dislocation density, and the hardening behavior, which enables insight into the evolution of the microstructure, texture, and mechanical properties at different length scales [1]. In the framework of CP applied in FE simulations, the microstructures generally can be classified into two types, real and artificial. Besides the applied theory, the meshed structure and the boundary conditions are the other two necessary aspects for the FE simulation. For a good FE model, any applied real or artificial microstructure should be well representative, which means its statistical estimations should match reality. Measurements based on in-situ tomography and electron backscattering diffraction (EBSD), including 3D EBSD [2,3,4], can deliver real microstructures. The Voronoi tessellations can present a random microstructure approximately (artificial structure) [5]. Within the class of Voronoi tessellations, the most intensively examined model is the Poisson Voronoi tessellations [5,6]. Concerning the application of Voronoi structures in simulations, one can referred to [7,8,9,10]. The manual work is enormous from the real microstructure with a few hundred grains to the meshed structure. Digital image processing can improve efficiency. For FE calculations, boundary conditions (BCs) are essential, which directly influence the accuracy of predicted results. For micromechanical simulations, homogeneous and periodic boundary conditions are the most applied two types of BCs. Recently, in-situ tests enabled the application of real boundary conditions, which is limited due to its availability.

For numerical multi-scale investigations of material deformation behaviors, it is vital to connect the same physical/mechanical properties across scales. The necessary force or the energy provoking the dislocation activations, such as movement initiation, motion, tangling, and annihilation, can take over this task. On the atomistic level, dynamic laws describe the dislocation mechanisms. The dislocation dynamics can be taken as the upper-scale model of atomistic simulations [11]. One aim of dislocation dynamics simulations is to link the dislocation properties on the elementary (nano) scale with those in continuous (micro or macro) models for the bulk material plasticity [11]. The description of the plasticity in terms of a collection of dislocation lines moving and interacting in a certain predefined way is the discrete dislocation dynamics (DDD) [12]. According to Schwarz [12], the DDD simulation models dislocation behavior on a continuum level since it treats the dislocations as continuum lines. DDD simulations can take account of contributions from dislocations far away, interface corrections, crystal anisotropy, etc. In other words, the results of DDD simulation can be directly applied to micromechanical simulations or be compared with those on the microscale. Homogenization can establish the relation of physical/mechanical properties on the global and micromechanical levels.

A deeper understanding of the particles’ double-edged effect on physical and mechanical properties is beneficial for both the academy and industry since it contributes to the thermal-mechanical processing design and microstructural control. The current main work consists of many-scale numerical investigations of the tensile deformation behavior of the commercial polycrystalline composite Ag/17vol.%-Tin-oxide (Ag/17vol.%SnO${}_{2}$) used in industry. The executed experiments were also many-scale-based [13,14]. Experimentally, the initial sharp fiber texture caused by the hot extrusion was reduced due to tensile loading. Still, a large number of Σ3-twins vanished after tension. Micro-macro two-scale simultaneous FE simulations emphasize micro deformation mechanisms providing comparable physical/mechanical properties with those from DDD simulations on the submicron scale. A short discussion is presented about the micro-macro transition zone material properties. The hardnesses and the local Young’s moduli distribution, obtained from nanoindentation tests, illustrate that the local inhomogeneity gradually decreased, approaching global homogenous properties. The elasto-viscoplastic material model based on CP describes the local grain deformation behavior. For 3D investigations, the special case of axisymmetry is preferred in the FE simulations since 2D real microstructures can be used as input and its calculation time is comparable with 2D simulations. FE results show deviations for the deformation behavior between Σ3-twins and the Ag phase. From micro down to (nano-micro) mesoscale, DDD achieves 3D simulations of dislocation motions. The resolved shear stress (RSS) evolutions are compared between DDD and FE simulations.

## 2. Materials and Experiments

### 2.1. Materials

Ag/SnO${}_{2}$ oxide dispersion strengthened (ODS) alloy is often used as electric contact material. Such commercial polycrystalline metal matrix composites (MMCs) are usually produced by two manufacturing processes, internal oxidation [15,16,17] and the standard powder metallurgical (PM) process [16]. It is possible to control the final oxide sizes using different SnO${}_{2}$ powders. The current work took Ag/17vol.%SnO${}_{2}$ (12wt.%SnO${}_{2}$) composites produced from the PM method as sample materials. The hot extrusion process is the commonly applied treatment in the industrial production of the Ag/SnO${}_{2}$ MMCs. This process introduces very strong initial fiber texture due to the recrystallization during cooling down and improves the mechanical behaviors of the oxide particle hardened MMCs. The green bodies produced by manufacturing processes were thermally extruded with an extrusion ratio of about 17:1 at 800 °C for one- to two-pass. After the final extrusion, the diameter of rods was 5 mm for all samples, and the mini cracks caused by the production process vanished [17]. For simplicity of discussion, only measured data used in this work will be shown in the following. A more detailed description of experiments and results are referred to Wasserbäch and Skrotzki [13] and Wasserbäch et al. [14].

### 2.2. Selected Material Data and Tensile Flow Curves

**Figure 1:**
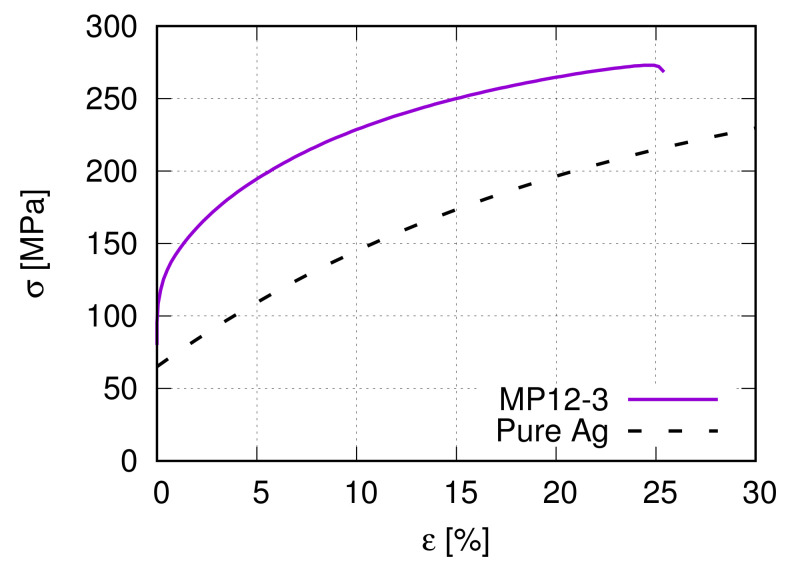
Global true stress–strain flow curves of the PM12-3 MMC [14] and the pure Ag (Table 1) under tension.

There are six different Ag/SnO${}_{2}$ samples, three from internal oxidation (IO) and three from PM manufacturing processes. Table 1 lists some material characteristic values for two Ag/17vol.%SnO${}_{2}$ composite (PM12-2 and PM12-3) and the pure Ag produced by the PM process. The names “PM12-2” and “PM12-3” in Table 1 are identical to the nomination in [13,14]. Figure 1 illustrates the measured stress–strain flow curves for the samples PM12-3 and pure Ag as given in Table 1.

### 2.3. Measured Σ3-Twins and Texture

**Figure 2:**
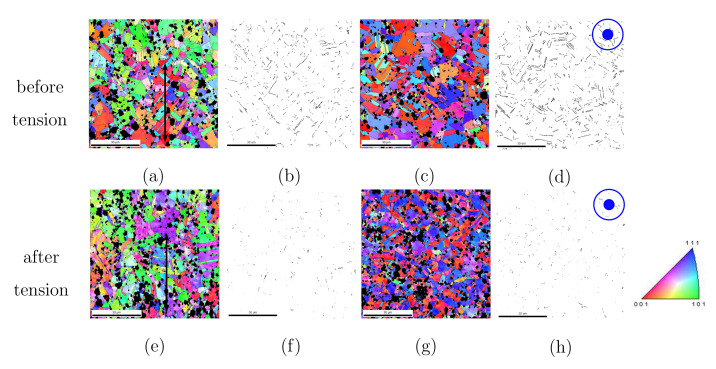
The Σ3-twins in PM12-3 composite from EBSD measurements: (**a**–**d**) after extrusion before tenion; (**a**) in the longitudinal direction with the arrow presenting the extrusion direction [13]; (**b**) Σ3-twin boundaries in (**a**); (**c**) in the transverse direction [13]; (**d**) Σ3-twin boundaries in (**c**); (**e**–**h**) analogous to (**a**–**d**), but at the material status after tension.

Due to shaping, extraordinary distortions, recrystallizations (during cooling down), and a large number of Σ3-twins were found. The observed microstructures, including Σ3-twins and textures, are due to recrystallization during or immediately after the hot extrusion process (at high temperature). The cooling process has a negligible effect on the Σ3-twins as well as on the textures. In our samples, the local true strain could reach the value of 500% caused by the hot extrusion. Figure 2 shows EBSD images and twin boundaries for the PM12-3 composite before and after the tensile loading, where the inclusion is given in black/dark color. The given color code in Figure 2 is valid for the Ag phase in all the results from EBSD images in this work. The blue dots in the upper right corner of Figure 2d,h means the extrusion or tension direction perpendicularly goes into this cross-section. Except for the morphology shown in black in EBSD images, all the mentioned EBSD measurements, e.g., grain orientations and textures, contain only the Ag phase. Figure 2a,c denote the material status after the extrusion (before tension) in the longitudinal and cross direction, respectively. Figure 2b,d illustrate Σ3-twin boundaries corresponding to Figure 2a,b. Figure 2e–h are analogous to Figure 2a–d for the material status after the tensile loading. The Σ3-twins amount strongly depends on the size of grains and particles [13,14]. The larger the size, the higher the amount of Σ3-twins. This work deals with annealing Σ3-twins. By comparison of Figure 2b,d,f,h, the amount of Σ3-twins reduced obviously due to tensile loading, even though the tensile loading direction is identical to the extrusion direction. After extrusion and before tension, the Σ3-twins possess about 13.0% and 23.4% (area fraction) in the longitudinal and cross-section (Table 1 last two columns). The Ag phase is very soft during hot extrusion, and the dislocations (in the Ag phase) can bypass the oxide particles. During cold-working (tension), the ceramic particles drastically affect the dislocation movement and distribution. It implies that the SnO${}_{2}$ particles significantly influence the Ag grain rotation during tension and, thus, affect texture evolution. Simultaneously, a large number of Σ3-twins are also destroyed. Sharp textures were formed in the Ag phase after the extrusion. The fiber texture sharpness was reduced after the monotonic tensile loading. The material underwent a tensile deformation during the extrusion shaping since the cross-section is reduced. Together with FE results, measured textures will be illustrated and discussed in Section 3.2.2.

### 2.4. Indentation Tests

**Figure 3:**
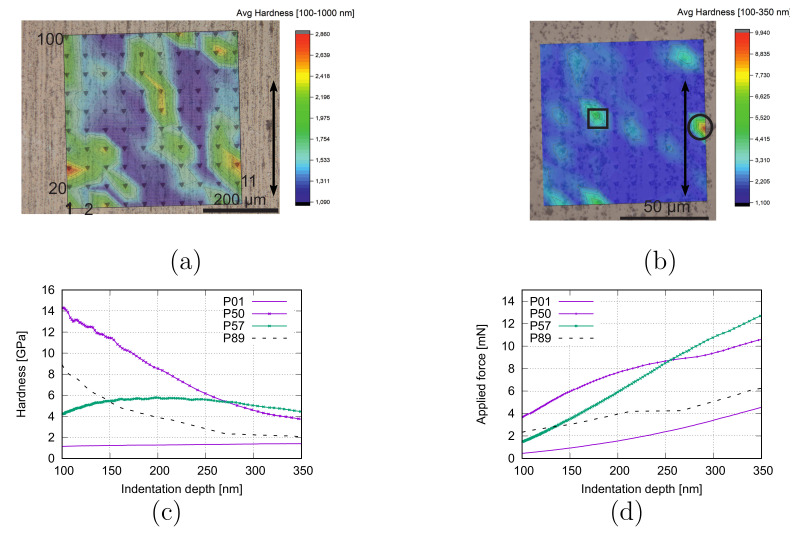
The nanoindentation measurement (HV0.3) of PM 12-2 MMC in the longitudinal section (100 measured points): (**a**) averaged values with 50 μm distance between two neighboring points and the arrow presenting the extrusion direction; (**b**) averaged values with 10 μm distance between two neighbor points and the arrow presenting the extrusion direction; (**c**) hardness value evolution according to indentation depth for some selected points in (**b**); (**d**) same as (**c**), but for applied forces.

Principally, our polycrystalline MMCs have local anisotropic and inhomogeneous characteristics. EBSD images can prove the former, referring to Figure 2 and [13,14]. For the latter, it can be shown by the Vickers nano hardness measurements. For the composite PM12-2, Figure 3a,b present the local hardness distribution (HV0.3) with the distance of 50 μm (HV-A) and 10 μm (HV-B) for neighboring two drilling points, respectively. Figure 3a also illustrates the nomination regulation for the measured points, also valid for Figure 3b. Both Figure 3a,b show heterogeneity for the local hardness property. The measured hardness is the mixed property from the Ag phase and particles due to particles’ small sizes for most drilling points. If the indentor tip meets the SnO${}_{2}$ particles, high hardness values appear. Figure 3c shows the hardness evolution according to the drilling depth from the same test as Figure 3b. The measured point marked with a circle in Figure 3b reached a maximum value of about 15 GPa at the drilling depth of about 67 nm (point 50). According to [18], the calculated hardnesses of SnO${}_{2}$ (from different methods) are around 10–13 GPa. The measured hardness of SnO${}_{2}$ is 3.68 GPa, according to [19]. The hardness variation is due to the different material status, e.g., lattice structure, initial state, or failure. The measured curve for point 57 marked in a rectangle in Figure 3b shows hardness increment according to indentation depth until about 230 nm and then decreases. While the curves for points 50 and 89 present decreasing behavior. Such phenomena are local morphology dependent, especially on SnO${}_{2}$ positions. Most curves cover a hardness range between ≈[1.0, 3.0] GPa. For the case in Figure 3a, the averaged hardness values cover a range of about [1.10, 4.23] GPa for the drilling depth [100, 350] nm and about [1.09, 2.86] GPa for the drilling depth [100, 1000] nm. For the HV-B test (Figure 3b,c), the averaged hardness range is about [1.10, 9.90] GPa for the drilling depth [100, 350] nm. The minimum value of 1.10 GPa should be near the pure Ag hardness. The Ag hardness of 927.4 ± 27 Nmm${}^{-2}$ (0.9274 ± 0.027 GPa) is presented in [20] and a measured hardness of about 1 GPa for pure Ag in [21]. From larger scale to smaller, the local heterogeneity should accordingly increase. The value range of the hardness in a smaller scale (Figure 3b) is more extensive than in a larger one (Figure 3a). Figure 3d is the same as Figure 3c, but for applied forces. Generally, the applied force increase according to drilling depth. However, their values also depend on the local morphology, especially SnO${}_{2}$ particles in the current case. By comparing Figure 3c,d, a tendency is that higher hardness positions are accompanied by higher applied forces, but deviations can appear, e.g., at about 150 nm and 250 nm drilling depth for the drilling points P50 and P57.

**Figure 4:**
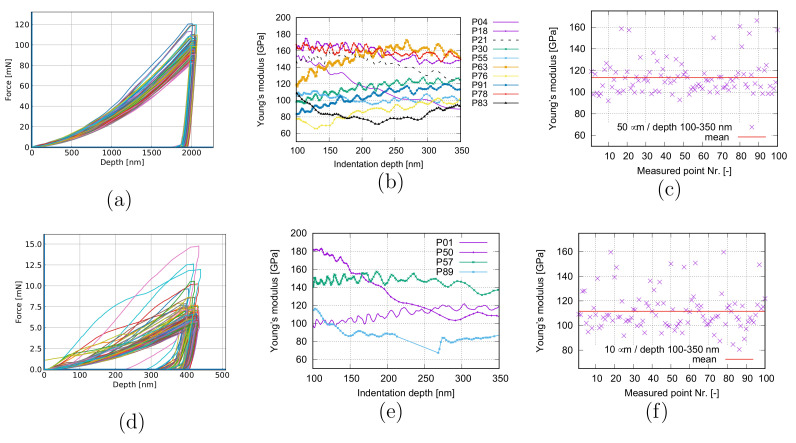
Applied force and Young’s modulus v.s. penetration depth and the recalculated mean values of Young’s moduli from the nanoindentation test (HV0.3) for PM 12-2 MMC in the longitudinal section: (**a**–**c**) and Figure 3a from the same test; (**d**–**f**) according to Figure 3b.

Figure 4 presents the applied load and Young’s modulus v.s. the penetration depth and the recalculated mean values of Young’s moduli. Figure 4a–c are obtained from the same test as Figure 3a, but the presented data correspond to different depth ranges. Figure 4d–f correspond to Figure 3b, and data are also extracted from the identical depth range. Through comparison of Figure 4a,d, the smaller scale (HV-B) presents more heterogeneity than the larger scale (HV-A). The stronger obvious fluctuation in Figure 4b results from the local morphology, and it should be mainly due to SnO${}_{2}$ distribution. If the area of the drilling point is larger (H-A), statistically, the possibility of covering the various amount of particles, including zero vol.% among measured points, reduces. It means the fluctuation tends to be smoother. The measured maximum Young’s modulus is about 259 GPa (HV-B) at about 40 nm of the drilling depth. It means that the drilling area should cover nearly 100% SnO${}_{2}$ particles. According to [20], including its cited works, this value is in [261, 268] GPa for pure SnO${}_{2}$. Figure 4b,d indicate that the heterogeneity of local Young’s modulus becomes milder according to the increment of the measured area. The deeper the depth, the more pronounced the tendency. Even though the mean values of the recalculated Young’s moduli (Figure 4c,f) cover approximately the same range (≈[70, 180] GPa), the scatter of data is more evident for the HV-B case. It means that the smaller the size, the higher the heterogeneity.

The measured local properties at different sizes will be used as input parameters for transition zones in micro-macro simultaneous simulations in the future. In many cases, the measured local stress, including the yielding stress, can be directly used in simulations. Generally, the study of establishing a relation between measured hardness (including elastic modulus) and the local (yield) stress is still in the investigation stage. It means no concrete recalculation form/ansatz exists between hardness value and the local stress, especially for the currently studied composite due to even less available data. The recalculation form, as mentioned above, should also vary from material to material. It means that our material cannot share the identical recalculation form (ansatz) for steels. Pavlina and Van Tyne [22] showed some examples for converting the hardness value to the stress value. With the measured force-depth, Young’s-modulus-depth data from nanoindentation tests, and the measured local strain data from digital image correlation tests, the subsequent step work will try to deduce and establish the relation between measured hardness and the local (yielding) stress.

## 3. Finite Element Simulation

For a good numerical prediction of the deformation behavior of polycrystalline materials such as ODS Ag/SnO${}_{2}$ MMCs in the current work, different micro-mechanisms should be considered. Such mechanisms include, e.g., the anisotropy, the interaction among grains, the dislocation activation, and the heterogeneity of local material properties. For ODS composites, the volume fraction of the strengthening phase is essential in determining the global flow behavior. In this work, all the mechanisms mentioned above and the local volume of the particle phase are considered in FE predictions (ABAQUS). Axisymmetric simulation is preferred since the 2D real microstructures can be directly applied in such FE calculations. It also possesses the advantage: calculation time as efficient as in 2D and the whole presentation of a given variable in three directions as in 3D. From tests, it is observed that the texture intensity reduced after the tensile loading, even though the tensile and extrusion direction are identical. The presence of the ceramic phase plays an essential role in the phenomenon mentioned above. Numerically, this work tries to figure out the main mechanisms of this texture evolution phenomenon and investigates the influence of Σ3 twins on the local deformation behavior. In FE simulations, the available theories to predict metallic material deformation behavior are more suitable for pure metals. For the case of metallic alloys/composite with second phase particles, much more study is necessary to reach a robust and sounded simulation process, including theories to describe their (metallic matrix and ceramic inclusion) deformation behavior in detail. The primary difficulty lies in the less knowledge of dislocation mechanisms in the presence of the ceramic phase.

### 3.1. Material Law

Under loading and on the micro-level, extraordinary large plastic deformation may appear, such as the Ag phase in our sample material. It requires a material model which can describe the local distortion and dilatation well. The elasto-viscoplastic material model from the CP is suitable to mechanically simulate the Ag phase deformation behavior on the microscopic level, which is in the category of the continuums mechanics. A user-defined subroutine (UMAT in ABAQUS, developed at the Mechanics Institute of Otto-von-Guericke-University Magdeburg, Germany) realized such FE simulations. This UMAT is already applied for different materials with non-identical crystal structures under different loading conditions [23,24,25,26,27,28,29,30,31]. The applied material model, including the homogenization method, is briefly described in Section 3.1.1 for discussion. The material deformation behavior on the macroscope obeys the von Mises plasticity, so-called “J${}_{2}$ theory”, in the two-scale simultaneous simulation. The macroscopic material behavior under loading is not interested in the current work, but the mechanisms on the microstructures. The utility of the macrostructure is to take over the BCs and let the microstructure be free of it. It is the solution to avoid the high homogenized (global) stress in the case of homogenous BCs directly applied to the real microstructure.

For two-scale FE simulations, the transition zone connects the local meshing on the μm level and the global meshing on the mm level. It requires that its property, on the one hand, provides a realistic environment for the microstructure and, on the other hand, is matchable to the overall material behavior. To the authors’ knowledge, the scale bridging mechanism is a field being under research, and no well-proved methods are available. An ideal case would be a seamless integration of analysis attributes, such as multiple overall strain loading. Concerning this topic, some studies can be found in [32,33]. Under the precondition that the deformation behavior of the transition zone is not the emphasis, our consideration is to experimentally find the local elastic properties at the various measured size and randomly assign these achieved values to the corresponding subregions in the transition zone. Figure 3 and Figure 4 present such local properties at different sizes obtained from nano indentation tests. However, the local yield stress, the parameter directly applicable in the transition zone, cannot be deduced from available test data. Still, no seamless integration algorithm is available for the currently studied composite in a given time. Due to limited data and in this work, the microstructure is taken as a whole. It means the transition zone property should be comparable with the homogenized physical variables, such as stresses. The homogenized stress from the microscope is comparable with the global ones from the macroscope if the microstructure is well representative. In our FE simulation, the real microstructure includes 513 Ag grains and about 222 SnO${}_{2}$ particles in an 80 × 80 μm${}^{2}$ size. It is taken as being representative enough. More directly, the transition zone has the same material property as the macrostructure.

#### 3.1.1. Crystal Plasticity: Elasto-Viscoplasticity

##### Elastic Law

The deformation gradient tensor ***F*** is multiplicatively decomposed into

(1) $$\begin{array}{c} F=F_{e}P^{-1}=F_{e}F_{p}, \end{array}$$

where ***P*** indicates the plastic transformation [34,35] and $F_{e}$ is defined as $F_{e}=FP$. If $P^{-1}=F_{p}$ is identified, this ($F=F_{e}P^{-1}$) leads to the same decomposition suggested by Lee (1969) [36], i.e., $F=F_{e}F_{p}$. The elastic distortion, dilatation, and rotations including rigid body rotations can be embodied in $F_{e}$. The plastic incompressibility implies $det(F_{p})=1$. $F_{p}$ consists of crystallographic slips along the slip system ($d_{\alpha },n^{\alpha }$) with the slip direction $d_{\alpha }$ and the slip plane $n^{\alpha }$. A finite anisotropic linear elastic law is used, in which the 2nd Piolar-Kirchhoff stress tensor $T^{2PK}$ is a function of the Green strain tensor ***E***. To achieve its time-independent relation after yielding, it is convenient to describe the stress in an undistorted state ($T_{e}^{2PK}$)

(2) $$T_{e}^{2PK}=\tilde{K}\left[E_{e}\right]$$

with $\tilde{K}$ denoting the time-independent elasticities with a lattice vector ${\tilde{g}}_{i}$

$$\tilde{K}={\tilde{K}}^{ijkl}{\tilde{g}}_{i}⊗{\tilde{g}}_{j}⊗{\tilde{g}}_{k}⊗{\tilde{g}}_{l}$$.

The time-dependent current elasticities has the form

$$K=P★\tilde{K},$$

where ★ presents the Rayleigh product. The stress tensor in this law is related to the Cauchy stress (true stress) tensor σ

(3) $$\begin{array}{ccc} T_{e}^{2PK} & = & \det\left(F\right)P^{-1}F^{-1}\sigma F^{-T}P^{-T}\\ \; & = & \det\left(F_{e}\right)F_{e}^{-1}\sigma F_{e}^{-T} \end{array}$$

The Kirchhoff stress tensor $T_{e}^{K}$ is related to $T_{e}^{2PK}$ as $T_{e}^{K}=F_{e}T_{e}^{2PK}F_{e}^{T}$.

##### Flow Rule

The flow rule is taken from the finite crystal viscoplasticity theory, in which the time evolution of ***P*** is specified in terms of the shear rate ${\dot{\gamma }}_{\alpha }$ and the Schmid tensors $M^{\alpha }$ with a certain slip system α. ${\dot{\gamma }}_{\alpha }$, the Schmid resolved shear stress $\tau^{\alpha }$ and $M^{\alpha }$ are given as:

(4) $$\begin{array}{ccccccc} {\dot{\gamma }}_{\alpha } & = & {\dot{\gamma }}_{0}\;\mathrm{sgn}\left(\tau_{\alpha }\right){\left|\frac{\tau_{\alpha }}{\tau_{\alpha }^{C}}\right|}^{m},\;\tau_{\alpha } & = & \tilde{K}T_{e}^{2PK}\cdot {\tilde{M}}^{\alpha }\approx T_{e}^{2PK}\cdot {\tilde{M}}^{\alpha },\;{\tilde{M}}^{\alpha } & = & {\tilde{d}}_{\alpha }⊗{\tilde{n}}^{\alpha }, \end{array}$$

respectively [37]. ${\dot{\gamma }}_{0}$ and $\tau_{\alpha }^{C}$ corresponds to the reference shear rate and the critical resolved shear stress. For a given spatial velocity gradient $L:=\dot{F}F^{-1}$, the flow rule can be formulated in terms of $F_{e}$,

(5) $$\begin{array}{ccccc} \dot{F_{e}}{F_{e}}^{-1} & = & L-F_{e}\tilde{k}\left(T_{e}^{\prime },\tau_{\alpha }^{C}\right){F_{e}}^{-1},\;\tilde{k}\left(T_{e}^{\prime },\tau_{\alpha }^{C}\right) & =: & \sum_{\alpha =1}^{N}{\dot{\gamma }}_{\alpha }\left(T_{e}^{\prime },\tau_{\alpha }^{C}\right){\tilde{M}}^{\alpha }, \end{array}$$

with the Mandel stress tensor $T_{e}=F_{e}^{T}T_{e}^{K}F_{e}^{-T}$. Initially, it is taken $F_{e}\left(0\right)=Q_{0}\;\in SO\left(3\right)$ with the initial grain (crystal) orientation $Q_{0}=g_{i}\left(0\right)⊗e_{i}$. Here $g_{i}$ and $e_{i}$ present the lattice vectors and the orthonormal basis $\left\{e_{i}\right\}$, respectively. In this work, $Q_{0}$ is obtained from the experiment. It is a reasonable assumption that the slip systems of face-centered-crystal materials harden isotropically [38] such that only one critical resolved shear stress $\tau^{C}$ appears in Equation (5). Practically, all the Ag grains possess the same initial $\tau_{0}^{C}$ (initial value of $\tau^{C}$) and will have different $\tau^{C}$ values after yielding. Slip systems of silver are chosen as 〈110〉{111} [39,40], respectively.

##### Hardening Rule

The Kocks-Mecking hardening rule [38,41] is applied, which is a type of the Voce rule and emphasizes the dislocation growth, accumulation, and annealing mechanisms. In the current work, two different ansatzes are applied for the hardening rule. The first one is used in the Taylor model, which has prescribed strains during the whole loading process. An algorithm can realize such a Taylor model, i.e., FE simulation is not necessary. It indicates that the material permanently hardens according to the load, and no interactions exist among grains. The first ansatz has the form

(6) $${\dot{\tau }}^{C}=\Theta_{0}\left(1-\frac{\tau^{C}}{\tau_{c}^{v}\left(\tau_{\alpha },\tau^{C}\right)}\right)\dot{\gamma }\left(\tau_{\alpha },\tau^{C}\right),$$

where

(7) $$\tau_{c}^{v}={\tau_{c}^{v}}_{0}{\left|\frac{\dot{\gamma }\left(\tau_{\alpha },\tau^{C}\right)}{{\dot{\gamma }}_{0}^{*}}\right|}^{\frac{1}{n}}.$$

$\Theta_{0}$ is the work hardening rate near the yielding point. ${\tau_{c}^{v}}_{0}=\frac{\alpha \mu }{\kappa \beta }$ (α,β: material constants; μ,κ: shear modulus, Boltzmann constant) is an input material parameter and can be identified from the experiment [38]. ${\dot{\gamma }}_{0}^{*}$ is a material constant ($10^{7}s^{-1}$). Other values are possible, but they should be kept in agreement with the order of magnitude expected from the dislocation theory [38]. The reference shear rate ${\dot{\gamma }}_{0}$ is a constant. The second ansatz is used for the FE simulations

(8) $$\tau^{C}\left(\gamma \right)=\tau_{0}^{C}+\left(\tau_{\infty }^{C}-\tau_{0}^{C}\right)\left(1-\exp\left(-\frac{\Theta_{0}}{\tau_{\infty }^{C}-\tau_{0}^{C}}\gamma \right)\right)+\Theta_{\infty }\gamma .$$

**Figure 5:**
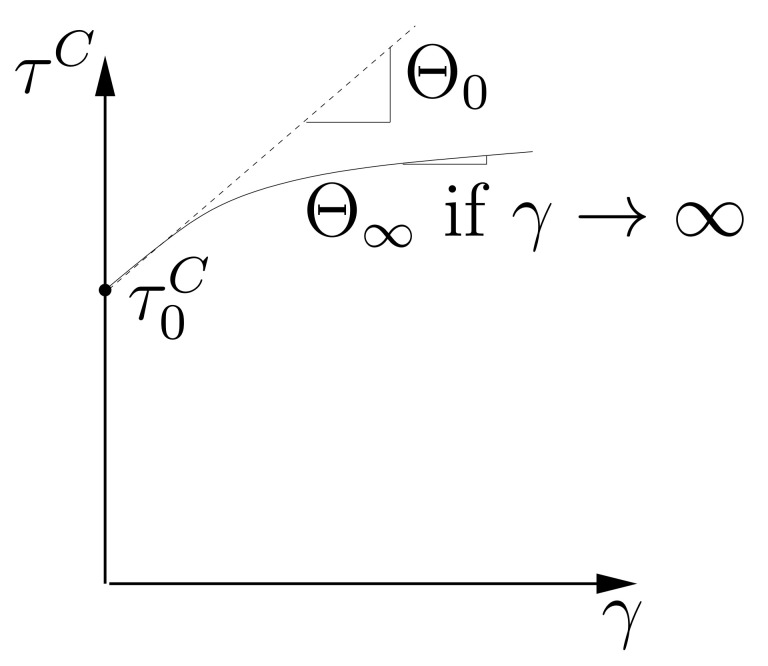
A sketch to show the work hardening rates $\Theta_{0}$ and $\Theta_{\infty }$.

$\Theta_{\infty }$ can be obtained by $\Theta_{\infty }=\lim_{\gamma \to \infty }\frac{\;d\tau^{C}}{\;d\gamma }$. γ is given by $\int_{0}^{t_{1}}\sum_{\alpha =1}^{N}\left|{\dot{\gamma }}_{\alpha }\right|\;dt$, where *t* presents the time and $t_{1}$ the time upper limit. $\tau_{0}^{C}$ is the shear stress for a given slip system at the yielding and $\tau_{\infty }^{C}$ is the one when γ→∞. Glüge et al. [42] have used the same hardening rule (ansatz (8)). The second ansatz is used to solve the problem that only one hardning rate ($\Theta_{0}$) is not enough to describe the hardening behavior far away from the yielding point in FE simulation. Figure 5 skematically shows positions of $\Theta_{0}$ and $\Theta_{\infty }$ in a σ−ε curve.

##### Homogenization

A representative volume element (RVE) is used in the current work in order to achieve the transition from the micro to macro variables. The macroscopic material variables are obtained through the homogenization of the corresponding micro fields. Based on the postulate of the work equivalence on the micro and the macro scale [43], the global first Piola-Kirchhoff stress (${\bar{T}}^{1PK}$) is related to the local one ($T^{1PK}$) by

(9) $${\bar{T}}^{1PK}=\frac{1}{V}\int_{V}T^{1PK}\;dV.$$

#### 3.1.2. Applied Structure, Meshing, and Boundary Conditions

**Figure 6:**
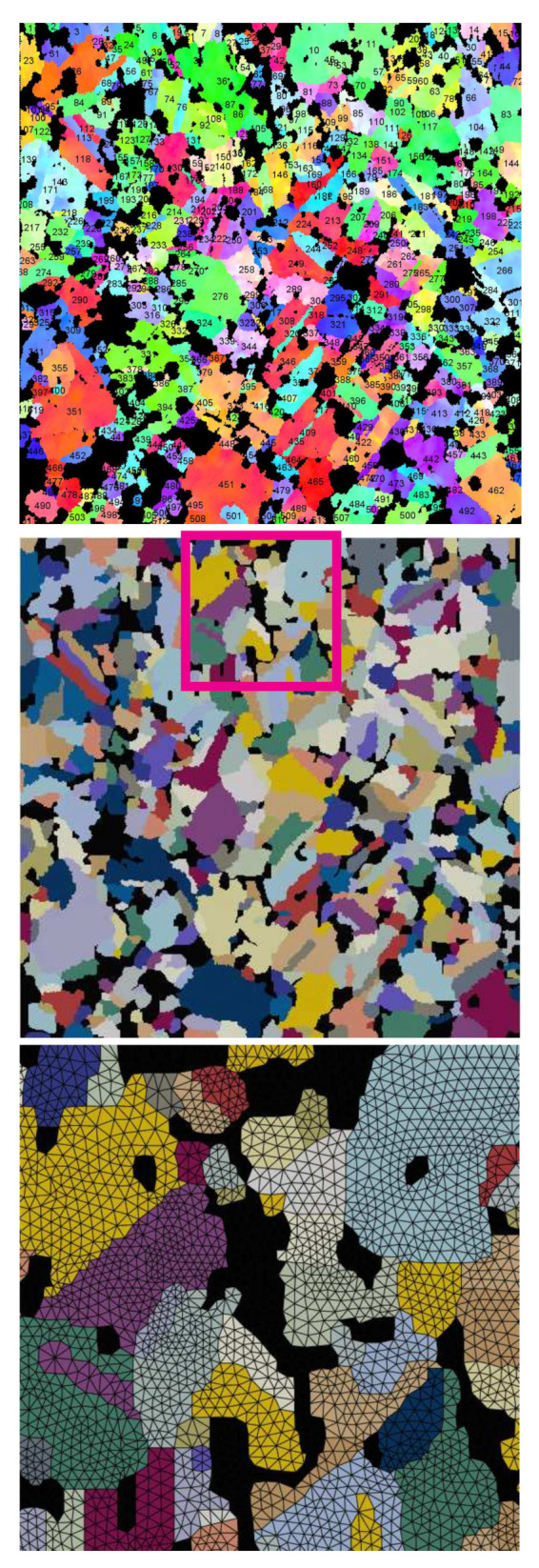
The real microstructure of Figure 2a with 513 Ag grains (**upper**); after pixel selection (**middle**); selected-region meshing (**lower**).

**Figure 7:**
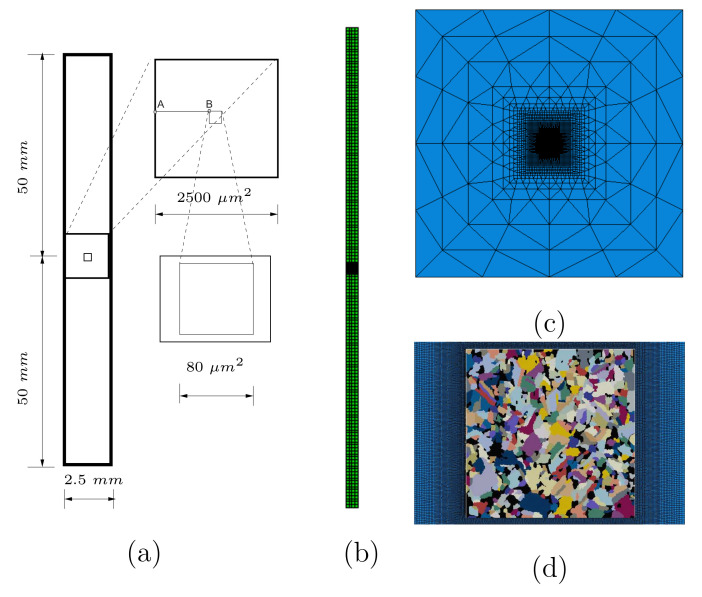
(**a**) Dimensions of micro, macrostructures, and the transition zone in the two-scale simultaneous FE simulation; (**b**) the meshing of the whole structure; (**c**) the meshing in the transition zone; (**d**) part of the meshing in the transition zone and the real microstructure with 513 Ag grains and about 222 SnO${}_{2}$ particles.

The real microstructure Figure 2a is selected for the axisymmetric FE simulation. Due to the fine pixel resolution (1080${}^{2}$ pixels in 80 μm${}^{2}$), Figure 2a can include thousands of Ag grains depending on the display choice of the grain size. Figure 6 (upper) with an individual grain covering no less than 45 pixels presents the same cut-out as Figure 2a. It leads to the minimum grain area of ≈0.25 μm${}^{2}$ (mean grain size ≈14.90 μm${}^{2}$). The microstructure (Figure 6, upper) includes 513 polycrystalline Ag grains and about 222 particles with ≈17 vol.% (the SnO${}_{2}$ 3D real volume fraction: 17 vol.%) and are taken as well representative. Figure 6 (middle) illustrates the microstructure after the pixel selection, ready for meshing. A slight manual modification of the total volume of SnO${}_{2}$ is done since the EBSD image possesses a little less volume fraction than the real one. The total volume fraction of the strengthening phase strongly affects the homogenized stress. Figure 6 (lower) denotes the geometrically adaptive meshed structure for the marked rectangle in Figure 6 (middle). This is done by the commercial software SimpleWare ScanIP [44], which only meshes 3D structures. The image Figure 6 (middle) is copied in the manifold to form an artificial EBSD tomography and meshed by SimpleWare ScanIP. To achieve the 2D meshing, a self-developed code is used to select the nodes, elements, and element sets (ABAQUS) on a proper surface in the 3D structure. An example of this process can be found in [7] (Figure 9 in [7]). “CAX” element type is used in micro-macro two-scale simultaneous axisymmetric simulations (ABAQUS). Figure 7a (left side) presents the macrostructural cross-section size corresponding to reality. The upper and lower sketches in Figure 7a (right side) present the dimensions for the transition zone and the microstructure, which also indicate the dimensions of Figure 7c,d, respectively. Figure 7b illustrates the meshing of the whole structure, where macro elements have a uniform edge length of 0.625 mm, totally four elements in the horizontal direction. Figure 7c shows the transition zone meshing approaching the macro elements. The meshing connection between the micro and transition zone is denoted in Figure 7d. For such meshing, the basic idea is to use triangle elements. It can realize the degradation of the total node number in a fixed size, i.e., increased node number from macro to microscale. For the case of a 2D structure applied in axisymmetric simulation, theoretically, the volume ratio of phases in the axisymmetric case (ABAQUS) is different from the area ratio in 2D. Practically, if the structure is positioned far away enough from the symmetric axis, the volume ratio converges to the area ratio. Four times of the structure edge length perpendicular to the symmetric axis should be enough. A simple mathematic proof and some numerical calculations can be found in [45]. The conclusion about the volume ratio convergence should be useful for all users encountering the same situation. In the current case, the distance between the microstructure and the symmetric axis ($\bar{AB}$ = 1210 μm in Figure 7a) is much larger than four times the size of microstructure (4 × 80 μm = 320 μm in the radial direction). It implies that the particle phase possesses about 17 vol.% in the FE simulation (microstructure in Figure 7d) as its area fraction in the microstructure.

For FE simulations, BCs always introduce more or less artificial effects for the simulated results since they set constraints for the degree of freedoms for edge nodes. These limitations for node displacements cause inaccuracies of the predicted material behavior. Usually, two types of BCs, homogeneous and periodic, are applied in FE simulations. Comparatively, periodic BCs (PBCs) improve the strong constraints of homogeneous BCs, since non-uniform displacements of boundary nodes are allowed in a periodic formulation for counterparts of edges/surfaces. PBCs require periodic structures, which cannot be fulfilled by the real microstructure (Figure 2a) in this work. To reduce the disturbances from BCs for the predicted micromechanical behaviors, two-scale simultaneous simulation is preferred. The macrostructure takes over the (homogeneous) BCs. It implies that the microstructure is free of BCs. This work only presents the numerical results from the micromechanical simulation.

#### 3.1.3. Parameter Identification

**Figure 8:**
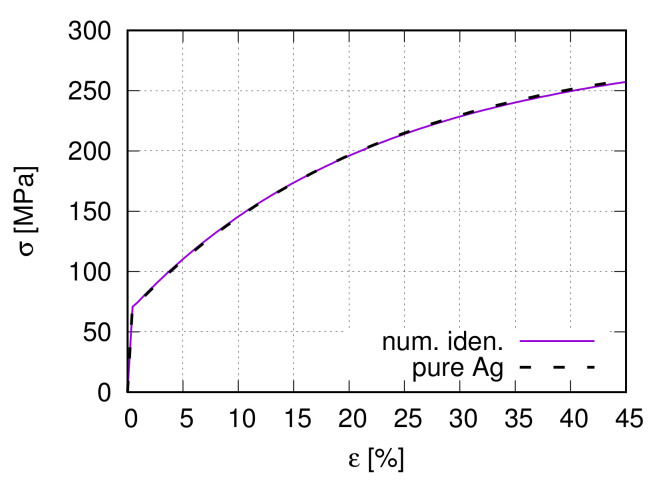
The experimental global flow curve and the numerical one predicted by the Taylor model for the parameter identification.

Experiment and literature provide a part of the input parameters. The linear elastically deformable SnO${}_{2}$ hard particle has Young’s modulus of 203 GPa and Poisson’s ratio 0.291, which are taken from Liebermann [46]. In Chang and Graham [47], Young’s modulus of Ag is 212.3 GPa (2.123 Mbar). For the Ag phase, the three independent components of the elasticity tensor $\tilde{K}$[48] are given in Table 2, which also lists some other constants used in the current work. For choices of ${\dot{\gamma }}_{0}$ and ${\dot{\gamma }}_{0}^{*}$ values, it is referenced to Kocks and Mecking [38]. The “Taylor model” (Section Hardening Rule) with prescribed strains for all considered grains delivers the remaining input parameters using the trial-and-error method. It means that such a model includes neither interactions between grains nor the grain size distribution (the mean size used). Such a process is also called inverse calculation. In inverse calculation, experimental measurements are usually applied for the calibration of the numerical results. Table 3 shows the final found values of $\Theta_{0}$, $\tau_{c0}^{v}$ and $\tau_{0}^{C}$. Using the data in Table 2 and Table 3, Figure 8 illustrates the experimental and numerical homogenized global flow behavior. The numerical curve is averaged from 1000 grains with randomly generated non-identical initial orientations.

However, previous experience [49] shows that the hardening rate is lower in the FE simulation than in the Taylor model due to grain-grain and grain-particle interactions. It causes a softer material behavior in FE calculations. In other words, the FE numerical prediction will not match the measured data so well as the Taylor model. To solve it, just a further work hardening rate $\Theta_{\infty }$ is necessary (Equation (8)), showing essential hardening effects at larger strains. For given $\frac{\;d\sigma_{0}}{\;d\varepsilon_{0}}$ and $\frac{\;d\sigma_{\infty }}{\;d\varepsilon_{\infty }}$, $\Theta_{\infty }$ can be calculated based on the experimental macroscopic stress–strain curves. Since $\Theta_{0}$ is already known from the Taylor model. $\Theta_{\infty }=5.39$ is fixed finally. A Taylor factor M = 3.0 is used during the deduction from the measured global Cauchy (true) stress σ to the resolved shear stress $\tau^{C}$. It is worth mentioning that only the experimental stress and strain data are necessary to obtain the entire input parameter set for the hardening ansatz given in Equation (8) if the parameter set is known for the hardening ansatz given in Equation (6).

Usually, the hard particle phase is assumed to be elastically deformable. Phase stress–strain curves from FE simulation (not shown here) showed that this assumption may lead to unrealistic high stress in the SnO${}_{2}$ phase. The observed numerical phenomenon is that the particles deform less in a microstructure with more volume fraction (about 23 vol.%) and deform more in the case with less volume fraction (about 17 vol.%). As a result, the homogenized stress–strain curves are nearly identical regardless of the particle volume fraction, which is not the truth. After analyzing, it should be caused by the numerical solution in solving the matrix of the whole system equations. It seems that by adjusting the particle deformation magnitude it is easy to satisfy all the requirements during searching solutions. On the other hand, the same material model (same user subroutine) is applied for different polycrystalline αFe-Cu composites [25], where the strengthening effect of the harder phase αFe is pronounced. It means the composite stress is non-identical for different compositions. Different material laws and homogenization methods applied for phases in the same microstructure also introduce more difficulty in the numerical solution searching. The assumption of being elastic-perfectly-plastic deformable can solve this problem. A stress upper bound with a value of 1250 MPa is found through FE simulations, i.e., independent of experimental measurements. A nano hardness test might provide valuable data for calibrating the correctness of the stress upper bound (1250 MPa). Further study is necessary to obtain knowledge of the relation between the hardness value and the yield stress (fracture stress).

### 3.2. Numerical and Experimental Result Comparison and Discussion

#### 3.2.1. Global and Local Stress-Strain Behavior

**Figure 9:**
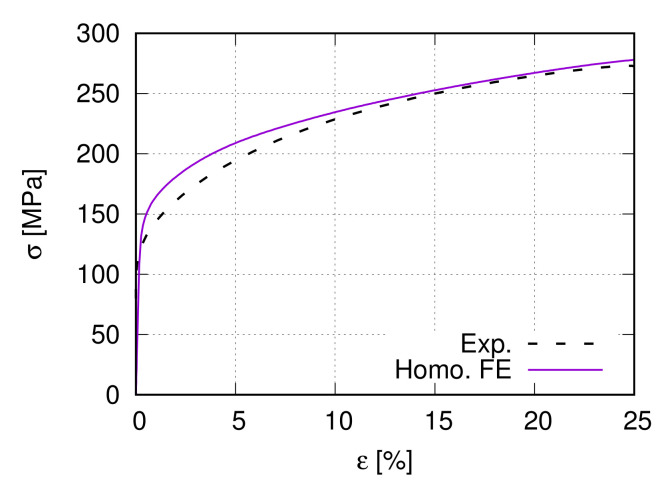
Comparison of the experimental [14] and homogenized σ−ε curves from FE prediction for PM12-3 Ag/SnO${}_{2}$ composite.

**Figure 10:**
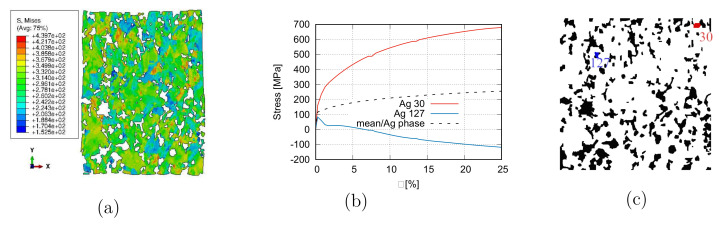
Micro-macro two-scale simultaneous FE simulation: (**a**) the von Mises stress distribution of the Ag phase and the deformed edges of the microstructure at 25% global strain; (**b**) the stress flow behavior according to the global strain: Ag phase mean value and two Ag grains possessing the maximum and the minimum stress at a given strain; (**c**) positions of the two grains in (**b**) together with black-colored particles.

Figure 9 illustrates the homogenized σ−ε curve from the two-scale FE prediction, which matches well the global flow behavior from the experiment. Figure 10a illustrates the von Mises effective stress distribution at 25% global strain, where non-uniformly deformed boundaries of the microstructure are apparent. It also presents the highly inhomogeneous stress distribution in the Ag phase, i.e., the flow behavior of individual Ag grains is quite different. The influential factors for results in Figure 10a are various, such as local morphology, interactions, compatibility, and grain orientations. The stress flow of two Ag grains, Ag-30 and Ag-127, are plotted in Figure 10b according to the global strain to demonstrate this inhomogeneity. Their stress–strain curves are located at the highest (Ag-30) and lowest (Ag-127) positions among the 513 Ag grains. In comparison, the average stress value of the Ag phase is also presented in Figure 10b. Figure 10c denotes their locations, Ag-30 in red and Ag-127 in blue, together with the black-colored particles in the microstructure. Both grains are not located on the microstructure boundaries.

#### 3.2.2. Texture Evolution

**Figure 11:**
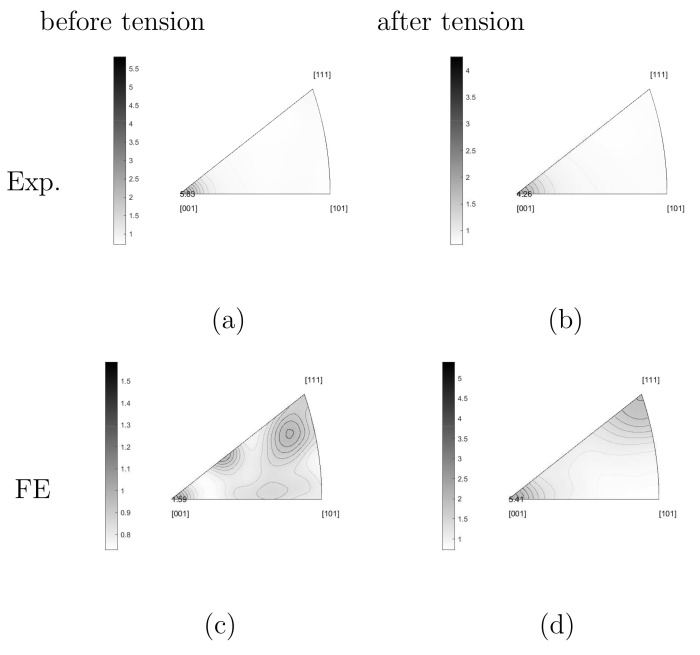
Standard inverse pole figures for the AgSnO${}_{2}$ PM12-3 composite: (**a**) EBSD measured initial texture after hot extrusion and before tension; (**b**) measured texture after tension; (**c**) initial texture for FE simulation (for experiment Figure 7a); (**d**) FE predicted textures after tension.

Standard inverse pole figures present the textures. Figure 11a,b illustrate the measured textures of the Ag phase before tension (after the hot extrusion) and after, respectively. The measured inverse pole figures in Figure 11a,b are calculated from different real microstructural cut-outs. Due to limited FE calculation capacity, the total number of Ag grains considered in the simulation is a few hundred. The commercial software OIM bound with the EBSD equipment can perform the task of grain coarsening and orientation averaging. Here, the grains covering less than 45 pixels are integrated into their neighbors. As a final result, 513 grains remain (testing result Figure 6a). It needs to point out that the original ESBD measurement (Figure 2a with thousands of Ag grains) shows a sharper fiber texture than after the recalculation of the grain size (Figure 6a with 513 Ag grains). Figure 11a results from the original measurement (Figure 2a) and Figure 11c from the one after the recalculation (orientation averaging) mentioned above (Figure 6a). In Figure 11c, there are 513 orientations presenting the mean orientations of individual grains. The difference between Figure 11a,c implies that the improvement of mapping the initial measured texture to the FE simulation is necessary. The improved result will be reported in another work. The experimental inverse pole figures presented in the current work cannot be the same as those in [13,14] since the currently shown results are from EBSD measurements and those in [13,14] from XRD measurements. On the other hand, the software (OIM) is used to calculate/plot the inverse pole figures in [13,14], but it cannot read the FE input data. The selected parameters for the data handling are identical in this work (Figure 11 and 14) but not completely the same as those in [13,14].

The inverse pole figure of the Ag phase shows the typical texture for face-centered-cubic (fcc) lattices, i.e., high fiber intensity in [001] and [111] direction after tension. As mentioned before, the material underwent a kind of tensile deformation in the extrusion process. The sample elongated further in the extrusion direction during the consequent tensile loading. From the test result, the fiber intensity reduced, especially in [001] direction (Figure 11a,b). For the PM12-3 composite, Σ3-twins occupies about 25 vol.% among all Ag grains before tension. After tension, this value is reduced to about 5 vol.%. The Σ3-twins should play a role in the texture evolution and further tests are necessary to make more concrete conculsion. The Ag phase texture is also affected by the SnO${}_{2}$ particles. Compared to pure Ag, the dislocation motions, including activation, tangling, stopping, diminishment, and other mechanisms, change drastically, accompanying the presence of the ceramic particles. Such dislocation mechanisms are not clear up to now. During hot extrusion, the Ag phase is very soft and the dislocations (in the Ag phase) can bypass the oxide particles. It means oxide particles’ effect on the texture evolution during hot extrusion is not as evident as during cold-working. However, during the room temperature tensile deformation, the microstructure and texture of the original as-extruded starting materials are modified. The microstructure and the texture upon cold-working strongly depend on the size of the dispersed oxides. Compared to fine particles with about 0.2 μm, larger particles (diameter d > 5 μm) significantly disturb the original microstructure and fiber texture present after hot extrusion. Systematic investigations of the influence of a second phase on the texture evolution upon cold-working by wire-drawing have been performed by Wassermann and co-workers [50,51,52,53] for several fcc metals. The main result is that the composite deformation texture strongly depends on the size and concentration of the oxide particles. TEM investigations revealed that high orientation gradients and dislocation density gradients are created around the larger particles [54], whereas a nearly random dislocation arrangement is observed in the case of small particles [55]. A similar dependence on the particle size was also observed in the case of cold-extruded Al (SiC) alloys [56]. Although our specimens (Ag/SnO${}_{2}$ ODS) are only tensile-deformed to a true strain of about 20% to 30%, a similar dependence of the texture on particle size is observed. Summarily, the ceramic particles (larger than 0.2 μm) destroy the initial texture during cold-working. As a result, the texture intensity reduced after tension. Further study is necessary to explain the exact mechanism which causes texture softening, not further strengthening.

Figure 11c presents the experimental texture of Figure 6 (upper) used as initial input for the FE simulation. Figure 11d presents the FE predicted texture after tension. It can capture the evolution of the [001] fiber intensity: the peak value in [001] direction after tensile loading. The relatively high-intensity values of other fibers at the initial state (Figure 11c) reduce and even vanish after tension (Figure 11d). It leads to the extinct high fiber intensity in [001] and [111] direction. The predicted inverse pole figure qualitatively matches the measured result. The simulated [001] fiber intensity possesses a higher value after tension than the initial one, but the experiment showed the other way round tendency. The FE simulation cannot predict the reduced sharpness of [001] fiber observed in the test. By comparing Figure 11a,c, the initial texture in the FE calculation has some deviation from the real one. It might cause the above-mentioned inaccurate prediction of the fiber intensity to a certain extent since initial texture also influences the simulation result. Bad mapping between experiment and simulation can cause the numerical prediction more away from the reality/experiment. The FE simulation considers the local morphology, grain orientations, and interactions. The special Σ3-twins orientations are also included since grain orientations in the FE calculation correspond to those in the experiment. However, there are no extra mechanisms to describe the Σ3-twins deformation behavior. Such mechanisms should be necessary for texture evolution. To the best of the authors’ knowledge, theories about twins’ deformation mechanisms combined with crystal plasticity are not well developed currently. The applied user-subroutine for the CP assumes isotropic hardening. In continuum mechanics, it means all the slip systems are activated simultaneously as soon as the CRSS reached. Possibly, this isotropic hardening is one reason causing those unmatch mentioned above. Besides extending the subroutine, further experimental investigation for the glide system activation mechanisms is needed to improve this isotropic hardening assumption. Since the parameter identification of non-identical CRSS for different glide systems is another necessary point. On the other hand, the presence of SnO${}_{2}$ particles plays a vital role in the Ag grain deformation. The dislocation motion mechanisms with the presence of the second ceramic phase should be quite different from those in pure metals. Currently, no very suitable material model exists to simulate the texture evolution in composites with a second ceramic phase. Much further work is necessary to numerically capture the phenomenon that the texture sharpness reduces after the tension, even though the tensile loading is in the same direction as the extrusion.

#### 3.2.3. Σ3-Twins Effect on Deformation Behaviors

**Figure 12:**
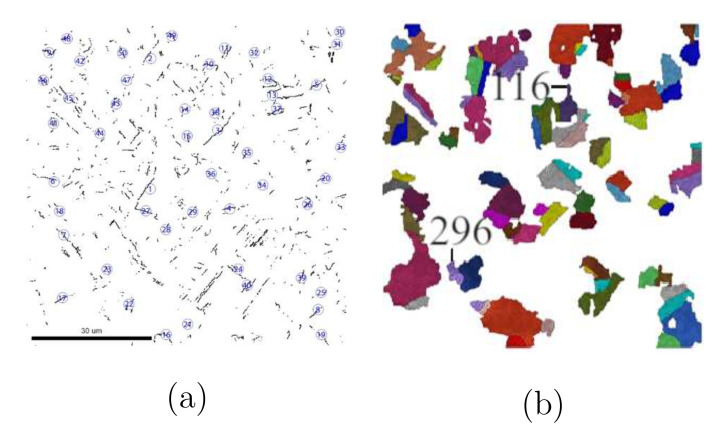
(a) Measured grain boundaries of Σ3-twins and selected 50 pairs of twins; (**b**) corresponding grains of the marked 50 pairs of Σ3-twins in (**a**).

**Figure 13:**
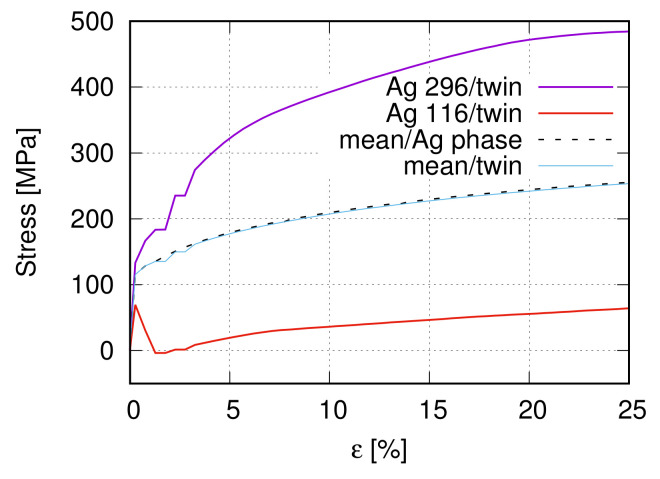
The stress flow behavior according to the global strain among the 50 pairs of Σ3-twins in Figure 12.

If the Σ3-twins play, more or less, a role in the fiber intensity reduction, another point would be whether their stresses also possess a distinguishable value than other grains or the mean value of the Ag phase. Due to limited measured data, these stress values are not available from tests. This work presents Σ3-twins stresses by FE predictions. Our applied crystal plasticity has the multiplicative decomposition of the deformation gradient tensor, where the elastic and plastic parts are coupled. Such a process can better describe the local deformation than decoupled elastic and plastic parts (additive decomposition of the deformation gradient tensor). It means the predicted results should also have relatively high accuracy. To show the stress flow behavior of Σ3-twins and compare the stress difference between Σ3-twins and other grains, 50 pairs of Σ3-twins are selected and numbered. Figure 12a illustrates the measured Σ3-twins’ boundaries and the numbered 50 pairs’ positions. Figure 12b represents their corresponding positions in the FE calculation. One grain can belong to more than one pair of these 50 pairs of Σ3-twins since some grains can compose Σ3-twins with more than one neighboring grain. Figure 12b includes 90 non-identical grains. Figure 13 illustrates the stress flow behavior of grains (mean stress inside one grain) according to the global strain. Curves marked as maximum (Ag-296) and minimum (Ag-116) indicate the highest and lowest stress value at a given strain. Positions of Ag-296 and Ag-116 are marked in Figure 12b. Figure 13 shows the grain-mean-stress v.s. global strain for the selected 50 pairs of Σ3-twins. By comparison of Figure 10 and Figure 13, it is obvious that individual grains in Σ3-twins result in a softer response to the outside loading than other grains. Statistically, the mean stress value of Σ3-twins should be used, which is comparable with the mean stress of the Ag phase (Figure 13). It is worth mentioning that Σ3-twins, unlike in the current case, can strengthen the material, as shown in [52]. In their case, the coherent boundary of Σ3-twins acts as hinders for dislocation movements. It means no atoms on the twin boundaries are arranged randomly, which can act as new dislocation activation sources. Through experiments, Konovalova et al. [57] showed that texture is strengthened upon an increased amount of the Σ3-twin boundaries for Cu, Ni, and Pd${}_{3}$ alloys. It is mentioned that no direct comparison of the Σ3-twins’ effect is available between the current work and Konovalova et al. [57] since the initial material status is not identical in both works.

**Figure 14:**
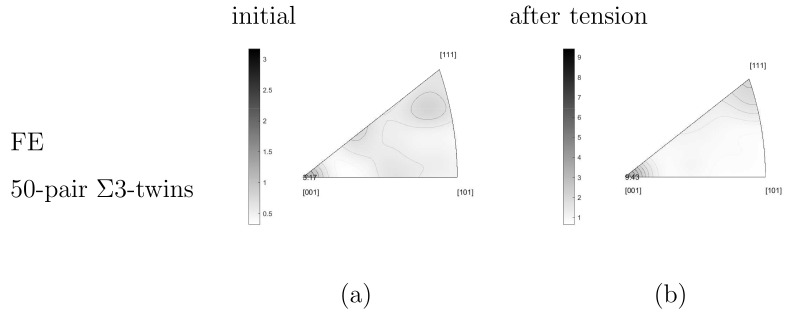
Standard inverse pole figures for the selected 50 pairs of Σ3-twins in Figure 12: (**a**) Measured initial texture after hot extrusion and before tension; (**b**) FE predicted textures after tension.

If the strength of Σ3-twins is slightly lower than but comparable with that of the Ag phase, the question is whether the texture evolution also shows the same tendency. Figure 14a,b from the FE simulation show the textures for the selected 50 pairs of Σ3-twins at the initial status and after the tensile deformation, respectively. By comparing the initial textures in Figure 11c and Figure 14a, the 50 pairs of Σ3-twins present a higher [001] fiber intensity than the Ag phase and also show a better match to the experimental result (Figure 11a). It means these Σ3-twins orientate themselves more intensively in [001] direction. As the initial state, besides [001] fiber, the texture of twins also shows other fibers with mild high-intensity. This texture characteristic is the same as the Ag phase (Figure 11c). The tensile loading enhances the [001] and [111] fiber intensity but reduces other fiber intensities so strongly that they nearly vanish. After loading, the [001] fiber possesses an intensity value increment of about 6.27 (9.43–3.17) calculated from the 50 Σ3-twins pairs and about 3.82 (5.41–1.59) calculated from the Ag phase. It means that Σ3-twins tend to rotate to the loading direction more strongly than other grains. Some pairs of Σ3-twins are not twins anymore after the tensile loading. The above-mentioned strong rotation should be the reason of the breakage of the twin pairs. As observed in the experiment (Figure 2), the Σ3-twins amount reduced after the tensile loading.

The selected 50 pairs of Σ3-twins present a little lower stress flow (according to global strain) than the Ag phase, but a sharper fiber texture. It seems that Σ3-twins possess more rigid body rotation than the average level of the whole Ag phase. In other words, such twins contribute to the strength less than others but more to the sharp texture. Possibly, it implies that rotations of other grains (not Σ3-twins) are the main reasons causing the texture intensity reduction observed in tests due to tension. This speculation requires more study.

## 4. Discrete Dislocation Dynamics Simulation and Result Comparison

EBSD real microstructures (Figure 2) showed Ag grains with and without tiny oxide particles inside them, i.e., not agglomerated at grain boundaries. DDD simulations concentrate on the latter case without fine particles in this work. Permanent dislocation movements originating from the atoms’ movement on the nanoscale cause metallic plastic behavior. The overall deformation behavior of MMCs is the result of coupled effects of various mechanisms on different scales. Accompanying the dislocation gliding, the RSS, including the CRSS, is essential for the deformation behavior. The homogenization technique links material deformation behavior on micro-macro scales. For a three-scale investigation, the next step is the coupling of mechanisms between nano-micro scales. Due to limited sources, the first trial study is performed on (nano-micro) meso scales in this work. The DDD describes the dislocations as discrete lines where the resolved shear stress evolves according to the dislocation density increments. The DDD simulations and the inverse modeling technique can establish a relation between local Young’s moduli and CRSSes. In such simulations, the initial randomly distributed dislocations are assumed as several finite-length segments. Following the interaction rules (e.g., Peach-Koehler forces, mobility laws) and considering mechanisms such as the cross-slip and the junction formation, a 3D simulation of the dislocation motion can be achieved. For in-depth information on DDD and its usage in simulation, we refer to [58,59]. Molnar et al. [60] present some previous work of the current authors on DDD simulations. A review of the DDD method can be found in [61] for numerical investigations of plasticity in crystals. In the current work, the aim is to show the influence of the grain orientation on its yielding stress and the increment of RSS. This implies that the difference in grain sizes is not considered in the DDD simulations. Here, the measured mean grain size and dislocation density (≈$0.50\times 10^{12}m^{-2}$) [13] are used. Other values of the initial density are also applied to show the initial dislocation density influence on the material response to loading.

**Figure 15:**
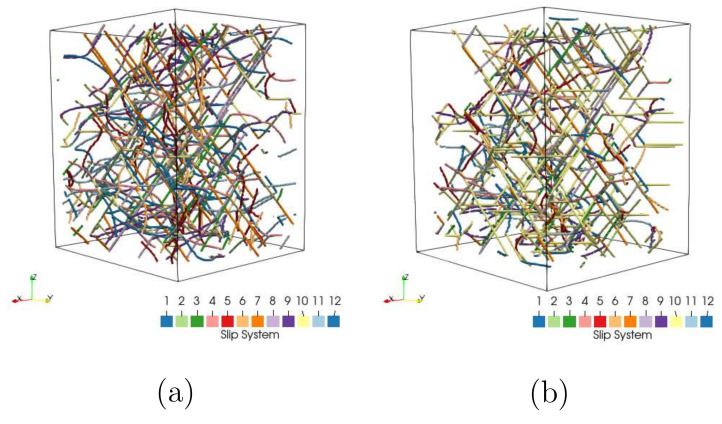
The dislocation morphology evolution predicted by a DDD simulation: (**a**) at the initial status; (**b**) after yielding.

Figure 15a,b denotes cut views of the dislocation distribution, also called dislocation morphology, at the initial and yielding stages, respectively. The dislocation density shows only minimum increment at yielding point compared to the initial state. The colors represent dislocations in all 12 fcc slip systems, including edge, screw, and mixed dislocations. Figure 15a illustrates the initial randomness and discrete dislocation lines. Figure 15b shows the influence of loading on the dislocation distribution pattern. The dislocation morphology evolution indicates that only the slip systems with the highest values of the Schmidt factor are activated by the external loading, whereas dislocations in systems perpendicular to the loading direction remain static.

**Figure 16:**
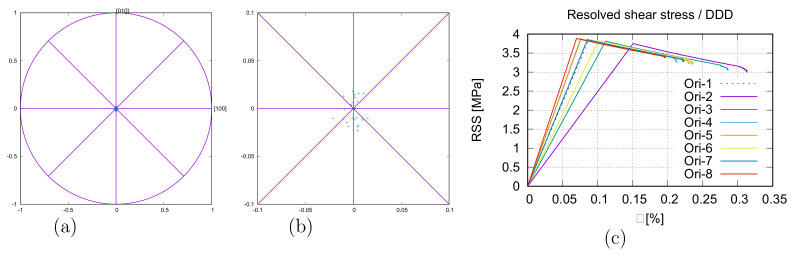
Eight DDD simulations: (**a**) the initial eight orientations projected to the loading axis (24 poles according to the symmetry); (**b**) zoom-in view for (**a**); (**c**) RSS-strain curves showing the inhomogeneous CRSS values (without multiplying the Taylor factor) and the variation of local Young’s moduli.

**Figure 17:**
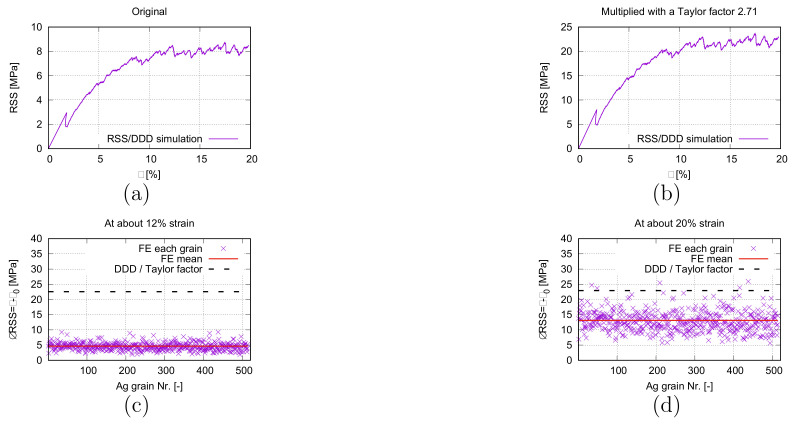
Predicted RSS and ΔRSS: (**a**) RSS flow behavior from DDD simulation; (**b**) the same as (**a**) multiplied by a Taylor factor to compare with FE results; (**c**) at 12% strain, ΔRSS ($\Delta \tau =\tau -\tau_{0}$) in 513 Ag grains and the Ag phase mean values from FE and DDD simulation (multiplied by a Taylor factor of 2.71); (**d**) analogous to (**c**), but at 20% strain.

Eight DDD calculations are performed with the measured mean initial dislocation density (0.5 × 10${}^{12}$$m^{-2}$ [13]) to denote the initial orientation effect on the yielding. Initial orientations around <100> are preferred since the very sharp initial texture (very high pole density near <100>) existed before tension. Figure 16a presents the projected pole positions in the {100} plane of the eight initial orientations, and Figure 16b is a zoom-in view. Due to symmetry, there are 24 poles in Figure 16a,b. The orientations are deduced from the measured grain orientations. Figure 16c illustrates the stress flow (RSS τ) behavior and Young’s modulus influenced by the eight-grain orientations mentioned above. The predicted CRSS values (RSS at yielding $\tau_{0}\approx 3.5–3.8$ MPa) did not present apparent deviations. By multiplying the Taylor factor (2.71 [14]), the CRSS will be $\tau_{0}\approx 9.5–10.3$ MPa. In contrast to the similar CRSS values, a relatively large distance for the yielding strain values is shown. It means the yielding strain presents stronger anisotropy than the yielding stress ($\tau_{0}$), and the local Young’s modulus also varies significantly (Figure 16c). The yield strains (in loading direction) lie between 0.07% and 0.15%, which corresponds to the common knowledge that most metallic materials yield before 0.2% strain (corresponding yield stress $R_{P_{0.2}}$ on the macroscope). Furthermore, the yielding strain also depends on the initial dislocation density (shown in Figure 17a). After yielding, the moving dislocation does not need the stress (τ) as high as $\tau_{0}$ at yielding to maintain its movement. The reason for this is the easy-glide causing stress decrement after yielding. The CRSS values ($\tau_{0}$) from the DDD simulation can be applied in a micromechanical FE simulation as local CRSS (initial value of $\tau^{C}$ in Equation (6) or $\tau_{0}^{C}$ in Equation (8)). It is pointed out that the Taylor factor should be considered during the linking between the DDD and the FE simulation since the former includes only a single grain orientation, but the latter deals with polycrystals. Concerning the $\tau_{0}$ values across scales, the measured macroscopic yielding stress ($R_{p_{0.2}}$) is ≈ 65 MPa in the loading direction. Theoretically, considering the Schmidt factor, the $\tau_{0}^{exp}$ should be ≤$\tau_{0}^{exp-max}$ = 0.5$R_{p_{0.2}}$ = 32.5 MPa in the dislocation moving direction. Here, $\tau_{0}^{exp-max}$ = 32.5 MPa is taken. The micromechanical simulation found $\tau_{0}^{num}$ = 35 MPa (Table 3) is close to the recalculated $\tau_{0}^{exp-max}$ = 32.5 MPa. It is a relatively good match between micro-macro scales. Based on stress–strain linear behavior before yielding (0.2% strain), the recalculated stress would be $\tau_{0}^{exp-assumed}\approx$ [11.73, 24.37] MPa (≈$\frac{0.07\%}{0.2\%}\tau_{0}^{exp-max}$ and $\approx \frac{0.15\%}{0.2\%}\tau_{0}^{exp-max}$, respectively), if the yield strain is in the range of ≈[0.07%, 0.15%] according to the DDD simulation. Here, it assumes a linear behavior for the measured yield stresses at strain lower than 0.2%. As mentioned above, $\tau_{0}$ predicted by DDD simulation ($\tau_{0}^{DDD}\approx$ [9.5, 10.3] MPa multiplied by a Taylor factor 2.71) is close to the lower bound of measured local yield stresses expected from the experiment ($\tau_{0}^{exp-assumed}\approx$ [11.73, 24.37] MPa). Due to these reasons, not all factors influencing the $\tau_{0}$ value are included in the DDD simulation here, such as the different grain sizes, the particle size, distribution, and the variation of the dislocation density in individual grains. These unconsidered factors cause deviations between DDD prediction and the experimentally expected. Furthermore, the dislocation density and the CRSS values from experiments are recalculated values, i.e., not from direct measurements. Some discrepancies may exist between recalculated values and real ones, which is difficult to be overcome due to the local randomness. Many further works are necessary to achieve a quantitatively good match of physical/mechanical properties across nano, micro, and macro scales, including meso scales.

Locally, the dislocation density has a discrepancy to the calculated mean values from the experiment. Another DDD calculation is performed with a higher dislocation density than the measured mean value to prove the significant effect of the dislocation density on the local yielding. Figure 17a from the DDD simulation (initial dislocation density $\approx 2.76\times 10^{12}$$m^{-2}$) illustrates the RSS-strain flow curve. Comparing Figure 16c and Figure 17a, the higher dislocation density influences the yielding strain much, but not the yielding stress. After yielding, the RSS value increased by ≈5.0 MPa at about 20% loading strain (Figure 17a). At 20% strain, the dislocation density is about $\approx 3.23\times 10^{12}$$m^{-2}$. To compare with the FE predicted RSS, a Taylor factor of 2.71 is used. Figure 17b is the scaled result of Figure 17a. Since the CRSS in FE is an input parameter, such comparison is performed for the ΔRSS ($\Delta \tau =\tau -\tau_{0}$) after the yielding. Figure 17c presents the numerical RSS increments from FE and DDD simulations at 12% loading strain. On the micro-level (FE), the RSS increment (Δτ) covers a range of ≈[1.9, 9.3] MPa presented as the mean value of each grain. The maximum value is about five times higher than the minimum one, which means high inhomogeneity exists on the microscale. The ΔRSS mean value for 513 Ag grains is ≈4.6 MPa at 12% global strain. The Δτ value is ≈8.3 MPa predicted by DDD simulation at 12% loading strain and this value is ≈22.5 MPa after scaling with a Taylor factor of 2.71. The FE predicted RSS increment is lower than the DDD predicted one. The reason for this is that the DDD simulation possesses linear stress increasing range and a higher hardening ratio after yielding than in the FE simulation. In FE calculation, the yielding is reached after one loading increment since the crystal plasticity is the emphasis. The linear elastic range is not comparable between FE and DDD simulations. As a result, the DDD predicted that ΔRSS is higher than FE did. The observed numerical behavior of the FE simulation is that the RSS rate ($\dot{\tau }$) was high at the lower strain (≈≤12% loading strain) and gradually decreased after ≈12% global strain. This behavior is also presented in Figure 9 for the global stress–strain comparison between FE calculation and measurement since the homogenized curve is higher than the experimental one before the 12% strain and nearly identical at 25% strain. It means $\Theta_{\infty }$ in Equation (8) contributes more and more to the hardening rate than $\Theta_{0}$ after 12% global strain. The above discussion implies that at larger strains (>12%), the predicted Δτ value from FE and DDD will be nearer and nearer to each other. Figure 17d is analogous to Figure 17c but at 20% global strain. The Δτ mean value of 513 Ag grains from the micromechanical prediction is about ≈13.12 MPa which is still lower than the DDD predicted ≈22.93 MPa from the DDD simulation. At 20% strain (Figure 17d), some high Δτ values from 513 Ag grains reach the Δτ level from the DDD simulation. Even higher local inhomogeneous stresses (RSS) in FE simulation are presented at 20% (Figure 17d) strain than at 12% (Figure 17c). DDD simulation presents very low RSS increment (approx. 22.93 − 22.56 = 0.37 MPa) from 12 to 20% loading strain.

## 5. Further Work

Concerning the link between micro and (nano-micro) mesoscale for multiscale simulations, no executable process is in sight at first. This is due to the limited time, since each microstructure includes at least hundreds of grains. One feasible method came to light after accomplishing the autonomous digital image segmentation using machine learning (ML) methods. The ML process resulted in a segmented image with eight colors. It means data for all 513 grains are parsed in eight colors (grain groups). These eight-grain groups can result in eight orientations. The eight resulting orientations can be used as the initial orientations for DDD simulations (not done in this work). The calculated CRSS values from DDD can be used as CRSS for the corresponding grains in micromechanical FE simulations and the calculation time is acceptable. The subsequent simulation in DDD would also include the effect of ceramic particles. The initial texture applied in FE simulations will be improved to match the measured values better. The new assignment of FE grain orientation will be based on the nearest neighbor method, the nearest measured point in the test to a given integration point in the FE meshing.

## 6. Conclusions

To numerically investigate material deformation behavior across three scales, commercial Ag/SnO${}_{2}$ polycrystalline composites are selected as sample materials. A remarkable phenomenon is observed for the measured texture: reduction of the [001] and [111] fiber intensity after tensile loading. Simultaneously, the volume fraction of Σ3-twins also decreased after tension. Generally, much more investigation is necessary to understand the dislocation motion mechanisms and the complex local interaction in the presence of the second ceramic phase. Another difficulty is the bridging for physical/mechanical properties predicted by divergent theories at various scales. For the numerical predictions in this work, the emphasis is put on the micromechanical deformation mechanisms described by the crystal plasticity theory. As the first step, micro-macro two-scale simultaneous FE prediction and the discrete dislocation dynamics simulation on the (nano-micro) mesoscale were performed under tension. Based on achieved results, the following conclusions can be drawn:

- The homogenized stress–strain flow behavior of the micromechanical FE simulation matches the measured global one very well;
- The FE prediction can capture the measured tensile texture volution characteristics: high [001] and [111] fiber intensity. The selected 50 pairs of Σ3-twins present a higher fiber intensity increment than the Ag phase. It means some Σ3-twins vanished after the tensile loading, which corresponds to the measured result (vol.% reduction of Σ3-twins);
- The Σ3-twins show a slightly softer hardening behavior compared to the Ag phase in the FE simulation;
- Based on the FE results, it let be deduced that Σ3-twins prefer to rotate to [001] and [111] fiber direction (twins disappear), and this preference caused other grains to rotate away from [001] and [111]. As a result, the overall [001] and [111] fiber intensity was deduced compared to the initial state (observation from the experiment);
- DDD-predicted local yielding strains (elastic moduli) strongly depend on the initial grain orientations and dislocation density. The dislocation morphology evolution indicates that only the slip systems with the highest values of the Schmidt factor are activated by the external loading, whereas dislocations in systems perpendicular to the loading direction remain static.
- At about 12% tensile strain, on the microscale, the ΔRSS values ($\Delta \tau =\tau -\tau_{0}$) among 513 considered Ag grains cover a range of ≈[2, 10] MPa and its mean value ≈4.6 MPa. A large discrepancy of the ΔRSS existed between the FE and DDD (Δτ≈ 22.5 MPa in DDD) simulations. However, at 20% loading strain, some FE predicted values of individual grains are comparable with the ΔRSS in DDD;
- From machine learning results for autonomous EBSD image segmentation, one performable method came to light to realize a multiscale simulation with the usage of experimental data: all considered hundreds of Ag grains are parsed into a few grain-groups due to the very sharp initial texture; for each grain-group, the resulting orientation and mean grain size (as input data for DDD simulation) can be calculated; the CRSS values ($\tau_{0}$) predicted by DDD simulations can be used as initial local yielding stresses for grains in the FE simulation (will be performed in the further work).

## Figures and Tables

**Table 1:** A list of some essential properties of the investigated MMC and the pure Ag produced by the PM process (LS: longitudinal section, CS: cross-section) [13].

Material	SnO${}_{2}$ vol.%	Ag D${}_{50}$ [μm]	SnO${}_{2}$ d${}_{50}$ [μm]	$R_{0.2}$ [MPa]	Σ3 [%] LS	Σ3 [%] CS
PM12-2	17	3.60	0.56 ± 0.16	118 ± 5	13.2	5.6
PM12-3	17	4.36	0.93 ± 0.20	106 ± 3	13.0	23.4
Ag 99.97	-	≈5	-	65	-	-

**Table 2:** A list of used input parameters for the Ag phase [38,48].

${\tilde{K}}_{1111}\left[\mathrm{GPa}\right]$	${\tilde{K}}_{1122}\left[\mathrm{GPa}\right]$	${\tilde{K}}_{1212}\left[\mathrm{GPa}\right]$|	${\dot{\gamma }}_{0}\left(s^{-1}\right)$	m	${\dot{\gamma }}_{0}^{*}\left(10^{7}\right)$	n
123.99	93.67	46.12	0.001	80	1.0	46.3

**Table 3:** Found out parameters using the Taylor model and the trial-and-error method (inverse modeling).

$\Theta_{0}[\mathrm{MPa}]$	$\tau_{c0}^{v}\left[\mathrm{MPa}\right]$	$\tau_{0}^{C}\left[\mathrm{MPa}\right]$
210	215	35

## Data Availability

Data are contained within the manuscript.
